# Supplementary material for: Lipopolysaccharide induces a downregulation of adiponectin receptors in-vitro and in-vivo
Source: PeerJ. 2015 Nov 19;3:e1428. doi: 10.7717/peerj.1428 (PMC4655095; doi:10.7717/peerj.1428)
Supplement: Data S1 [file peerj-03-1428-s001.docx]

| Incubation time | Group | 2^-ΔΔCT^ | +SEM | -SEM | p-value |
| --- | --- | --- | --- | --- | --- |
| 4 hours | Control | 1 | 0.106533 | 0.096276 |  |
|  | 0.1 μg/ml | 0.651574 | 0.088875 | 0.78208 | 0.044523 |
|  | 1 μg/ml | 1.107009 | 0.171281 | 0.14833 | 0.577524 |
|  | 5 μg/ml | 0.83702 | 0.127975 | 0.111004 | 0.334789 |
|  | 10 μg/ml | 0.870752 | 0.067074 | 0.062277 | 0.314486 |
| 24 hours | Control | 1 | 12.53022 | 0.926091 |  |
|  | 0.1 μg/ml | 0.904379 | 0.158907 | 0.135159 | 0.682949 |
|  | 1 μg/ml | 0.908568 | 0.176939 | 0.148098 | 0.626799 |
|  | 5 μg/ml | 0.99654 | 0.155679 | 0.134645 | 0.981832 |
|  | 10 μg/ml | 0.95705 | 0.091054 | 0.083144 | 0.707906 |

Figures 1 and 2

AdipoR1 gene expression in 3T3-L1 adipocytes following 0.1-10 µg/ml LPS treatment for 4 and 24 hours

AdipoR2 gene expression in 3T3-L1 adipocytes following 0.1-10 µg/ml LPS treatment for 4 and 24 hours

| Incubation time | Group | 2^-ΔΔCT^ | +SEM | -SEM | p-value |
| --- | --- | --- | --- | --- | --- |
| 4 hours | control | 1 | 0.216885 | 0.17823 |  |
|  | 0.1 μg/ml | 0.721798 | 0.183034 | 0.146009 | 0.272215 |
|  | 1 μg/ml | 0.387786 | 0.127606 | 0.096012 | 0.02309 |
|  | 5 μg/ml | 0.596668 | 0.098189 | 0.084314 | 0.066087 |
|  | 10 μg/ml | 0.255784 | 0.104501 | 0.07419 | 0.017648 |
| 24 hours | Control | 1 | 0.247877 | 0.198639 |  |
|  | 0.1 μg/ml | 0.961483 | 0.131424 | 0.11562 | 0.898072 |
|  | 1 μg/ml | 0.92445 | 0.147892 | 0.127495 | 0.77511 |
|  | 5 μg/ml | 1.194715 | 0.237475 | 0.198099 | 0.553701 |
|  | 10 μg/ml | 0.907519 | 0.139032 | 0.120562 | 0.698579 |

**Figures 3 and 4**

AdipoR1 gene expression in C2C12 myocytes following 0.1-10 µg/ml LPS treatment for 4 and 24 hours

| Incubation time | Group | 2^-ΔΔCT^ | +SEM | -SEM | p-value |
| --- | --- | --- | --- | --- | --- |
| 4 hours | Control | 1 | 0.088 | 0.080 |  |
|  | 0.1 µg/ml | 0.914 | 0.031 | 0.029 | 0.36 |
|  | 1 μg/ml | 0.989 | 0.039 | 0.037 | 0.91 |
|  | 5 μg/ml | 0.65 | 0.088 | 0.077 | 0.02 |
|  | 10 μg/ml | 0.79 | 0.06 | 0.056 | 0.06 |
| 24 hours | Control | 1 | 0.10 | 0.096 |  |
|  | 0.1 µg/ml | 1.15 | 0.083 | 0.077 | 0.27 |
|  | 1 μg/ml | 1.34 | 0.14 | 0.134 | 0.08 |
|  | 5 μg/ml | 1.19 | 0.16 | 0.147 | 0.32 |
|  | 10 μg/ml | 0.87 | 0.05 | 0.054 | 0.28 |

| Incubation time | Group | 2^-ΔΔCT^ | +SEM | -SEM | p-value |
| --- | --- | --- | --- | --- | --- |
| 4 hours | Control | 1 | 0.34 | 0.25 |  |
|  | 0.1 µg/ml | 1.05 | 0.09 | 0.08 | 0.85 |
|  | 1 μg/ml | 1.25 | 0.12 | 0.11 | 0.49 |
|  | 5 μg/ml | 0.76 | 0.11 | 0.101 | 0.44 |
|  | 10 μg/ml | 1.15 | 0.11 | 0.1 | 0.65 |
| 24 hours | Control | 1 | 0.07 | 0.07 |  |
|  | 0.1 µg/ml | 1.715 | 0.28 | 0.244 | 0.01 |
|  | 1 μg/ml | 1.14 | 0.14 | 0.12 | 0.36 |
|  | 5 μg/ml | 1.81 | 0.10 | 0.09 | 0.0001 |
|  | 10 μg/ml | 1.185 | 0.06 | 0.065 | 0.106 |

## C2C12 Myocytes: Adiponectin receptor R2 gene expression following LPS

Figures 5 and 6

Adiponectin gene expression in 3T3-L1 adipocytes following 1-10 µg/ml LPS treatment for 4 and 24 hours

| Incubation time | Group | 2^-ΔΔCT^ | +SEM | -SEM | p-value |
| --- | --- | --- | --- | --- | --- |
| 4 hours | control | 1 | 0.381852 | 0.276333 |  |
|  | 0.1 μg/ml | 1.406393 | 0.26345 | 0.221886 | 0.210711 |
|  | 1 μg/ml | 0.406596 | 0.248782 | 0.154344 | 0.153832 |
|  | 5 μg/ml | 0.223498 | 0.319371 | 0.131484 | 0.161381 |
|  | 10 μg/ml | 0.148137 | 0.04888 | 0.036753 | 0.002181 |
| 24 hours | Control | 1 | 0.725798 | 0.420558 |  |
|  | 0.1 μg/ml | 0.669737 | 0.100179 | 0.087144 | 0.186943 |
|  | 1 μg/ml | 0.593231 | 0.352092 | 0.220953 | 0.483881 |
|  | 5 μg/ml | 0.860551 | 0.349309 | 0.248457 | 0.83982 |
|  | 10 μg/ml | 0.565135 | 0.368188 | 0.222941 | 0.419607 |
