# Supplementary material for: Lipopolysaccharide induces a downregulation of adiponectin receptors in-vitro and in-vivo
Source: PeerJ. 2015 Nov 19;3:e1428. doi: 10.7717/peerj.1428 (PMC4655095; doi:10.7717/peerj.1428)
Supplement: Data S2 [file peerj-03-1428-s002.docx]

Supplemental files

Table 1

Relative change adiponectin receptor gene expression in mouse tissue depots 4 hours and 24 hours after treatment with LPS 25 mg/kg. Gene expression was determined by real-time PCR. Relative gene expression was calculated using the 2-ddCt method and p<0.05 was considered significant. (* denotes p<0.05). The reference group for calculations was the control group (ip saline injection) and housekeeping gene was β-actin. (SEM: standard error of mean, EF: Epididymal fat, PRF: Peri-renal fat, SCF: Subcutaneous fat)

| 4 hours | AdipoR1 |  |  | p value | AdipoR2 |  |  | p value |
| --- | --- | --- | --- | --- | --- | --- | --- | --- |
|  | 2- ddCt | +SEM | -SEM |  | 2- ddCt | +SEM | -SEM |  |
| Liver | 0.544 | 0.084 | 0.073 | 0.05 | 0.371 | 0.084 | 0.069 | 0.008* |
| Muscle | 0.102* | 0.109 | 0.052 | 0.017* | 0.162 | 0.43 | 0.11 | 0.39 |
| EF | 0.671 | 0.260 | 0.187 | 0.48 | 0.543 | 0.173 | 0.132 | 0.24 |
| PRF | 0.627* | 0.005 | 0.004 | 0.0087* | 0.231 | 0.067 | 0.052 | 0.0043* |
| SCF | 0.821 | 0.270 | 0.203 | 0.81 | 0.348 | 0.095 | 0.074 | 0.041* |

| 24 hours | AdipoR1 |  |  | P value | AdipoR2 |  |  | P value |
| --- | --- | --- | --- | --- | --- | --- | --- | --- |
|  | 2- ddCt | +SEM | -SEM |  | 2- ddCt | +SEM | -SEM |  |
| Liver | 0.614 | 0.053 | 0.049 | 0.09 | 0.650 | 0.142 | 0.117 | 0.148 |
| Muscle | 0.509* | 0.038 | 0.036 | 0.01* | 0.448 | 0.083 | 0.069 | 0.05* |
| EF | 1.01 | 0.316 | 0.241 | 0.47 | 0.852 | 0.213 | 0.17 | 0.55 |
| PRF | 0.801 | 0.145 | 0.122 | 0.27 | 0.657 | 0.187 | 0.145 | 0.198 |
| SCF | 0.824 | 0.211 | 0.168 | 0.47 | 1.058 | 0.312 | 0.241 | 0.98 |
